# Importance of ipsilateral residual hearing for spatial hearing by bimodal cochlear implant users

**DOI:** 10.1038/s41598-023-32135-0

**Published:** 2023-03-27

**Authors:** Mathew Thomas, John J. Galvin, Qian-Jie Fu

**Affiliations:** 1 Department of Head and Neck Surgery, David Geffen School of Medicine, University of California, Los Angeles, 650 Charles E. Young Drive South, CHS 62-113, Los Angeles, CA 90095-1737 USA; 2 House Institute Foundation, Los Angeles, CA 90057 USA

**Keywords:** Human behaviour, Perception

## Abstract

Bimodal cochlear implant (CI) listeners have difficulty utilizing spatial cues to segregate competing speech, possibly due to tonotopic mismatch between the acoustic input frequency and electrode place of stimulation. The present study investigated the effects of tonotopic mismatch in the context of residual acoustic hearing in the non-CI ear or residual hearing in both ears. Speech recognition thresholds (SRTs) were measured with two co-located or spatially separated speech maskers in normal-hearing adults listening to acoustic simulations of CIs; low frequency acoustic information was available in the non-CI ear (bimodal listening) or in both ears. Bimodal SRTs were significantly better with tonotopically matched than mismatched electric hearing for both co-located and spatially separated speech maskers. When there was no tonotopic mismatch, residual acoustic hearing in both ears provided a significant benefit when maskers were spatially separated, but not when co-located. The simulation data suggest that hearing preservation in the implanted ear for bimodal CI listeners may significantly benefit utilization of spatial cues to segregate competing speech, especially when the residual acoustic hearing is comparable across two ears. Also, the benefits of bilateral residual acoustic hearing may be best ascertained for spatially separated maskers.

## Introduction

For many cochlear implant (CI) users, relaxed candidacy criteria, modified electrode designs, and improved surgical techniques have preserved residual acoustic hearing in non-implanted and/or implanted ears. Residual acoustic hearing provides detailed low-frequency information that can greatly benefit CI users under challenging listening conditions. For listeners with one CI, combined acoustic and electric hearing can be categorized into three listening conditions: (1) *Bimodal*: electric stimulation in one ear and acoustic stimulation in the other ear; (2) *EAS*: electric stimulation and acoustic stimulation in the same ear; and (3) *BiEAS*: electric stimulation in one ear and acoustic stimulation in both ears.

Bimodal listening has been shown to significantly improve speech and music perception relative to performance with the CI alone^1–13^. However, some CI users do not experience significant benefits with Bimodal listening^14–16^ and others even experience interference^11,13,17,18^. Performance with EAS has been shown to be better than that with the CI alone, with even greater benefits observed for BiEAS^19^. The data from these previous studies indicate that the benefits of combined acoustic and electric hearing can be highly variable, possibly due to differences among individual CI users’ ability to combine acoustic and electric stimulation patterns within or across ears^20^.

In addition to providing useful low-frequency temporal fine-structure information, residual acoustic hearing in the non-implanted ear may provide some amount of binaural hearing in combination with the implanted ear. This low-frequency binaural hearing may be useful for sound localization and segregation of sound sources in space. However, for bimodal CI users, residual hearing in the non-implanted ear seems to have limited benefit for sound localization^21^ and segregation of spatially separated target and masker speech^22^. Dorman et al.^21^ found that sound localization scores were near chance level for bimodal CI users. Similarly, recent studies found little, no, or even negative spatial release from masking (SRM, defined as the performance difference in recognition of target speech between spatially separated and co-located maskers) for Bimodal CI listeners when head shadow effects were minimized by using symmetrically placed maskers^22,23^.

Normal-hearing (NH) listeners can use various spatial cues, such as inter-aural time differences (ITDs), inter-aural level differences (ILDs), and/or some other binaural process (e.g., inter-aural coherence)^24,25^ to better detect or recognize a target in the presence of competing sounds. For segregation of spatially separated speech, there are several benefits when listening with two ears over a single ear: head shadow (where the target-to-masker ratio, or TMR, is better in one ear than the other), binaural summation (where the redundant binaural representation allows for better segregation of the target), and binaural squelch (where the addition of the ear with the poorer TMR improves performance over the better ear alone)^26^. In general, bimodal hearing may not preserve important ITDs and/or ILDs due to timing differences (e.g., different compression time constants) between the acoustic hearing ear and the CI ear^27–30^. For many Bimodal CI patients, residual hearing in the contralateral ear provides only limited information due to the severity of underlying hearing loss. Without high-frequency audibility in the non-implanted ear, Bimodal listeners cannot benefit from ILD cues in the higher frequency region; for CI users, ILDs are the dominant cue for spatial perception. Firszt et al.^31^ reported limited bimodal benefit for spatial perception in Bimodal listeners with sloping or severe-to-profound hearing loss in the high-frequency region (i.e., the vast majority of Bimodal CI patients), compared to Bimodal listeners who have broadband audibility in the non-implanted ear.

Different from Bimodal CI patients, bilateral CI patients seem able to benefit from some spatial cues for sound localization^21^, possibly due to the availability of high-frequency ILDs. While bilateral CI users may be able to use ILD cues for sound localization, the bilateral benefit for segregation of spatially separated target and masker speech are limited. This is especially true when head shadow effects are minimized by using symmetrically placed maskers^22,23^. The lack of SRM in these studies may be partly driven by possible inter-aural frequency mismatch due to different insertion depth and uneven nerve survival across ears. Indeed, recent bilateral CI simulation studies have shown that inter-aural tonotopic mismatch may negatively affect utilization of spatial cues^32–34^. Thomas et al.^34^ found that minimizing the inter-aural mismatch may significantly increase SRM, possibly because that inter-aural frequency matching may improve inter-aural coherence.

Similarly, Bimodal CI users’ difficulties in utilizing spatial cues may be partly due to the tonotopic mismatch between the acoustic input frequency and the electrode place of stimulation in the cochlea. In clinical fitting of Bimodal CI users, the lowest acoustic input frequency is typically much lower than the characteristic frequency associated with the most apical electrode position^35^, resulting in some degree of tonotopic mismatch in the CI ear. Simulation studies have shown that tonotopic mismatch negatively affects the integration of acoustic and electric stimulation, regardless of whether residual acoustic and electric hearing were combined within an ear or across ears^36,37^. However, little is known about the effects of tonotopic mismatch on SRM for Bimodal listening.

One approach to reduce tonotopic mismatch in the CI is to adjust the input acoustic range to match the characteristic frequencies associated with the electrode positions. For most bimodal CI users, this would involve increasing the lowest acoustic input frequency, truncating all information below the adjusted input frequency. The loss of low-frequency speech information in one CI ear with a tonotopically matched frequency allocation may be less deleterious for Bimodal CI users since low-frequency speech information would be available with the contralateral acoustic hearing. Fowler et al.^38^ evaluated the effects of adjusting the low cutoff frequency (LCF) of the CI for CI-only and Bimodal CI listeners. They found that for the CI-only group, increasing the LCF reduced speech performance in both quiet and in noise. Similar results were observed for Bimodal listeners with limited acoustic hearing in the non-implanted ear (thresholds > 60 dB HL at 250 and 500 Hz). For Bimodal listeners with better hearing in the non-implanted ear (thresholds < 60 dB HL at 250 and 500 Hz), word recognition in quiet improved as the LCF was increased. Increasing the LCF likely reduced the tonotopic mismatch in the CI ear due to the relatively shallow electrode insertion depth.

While contralateral acoustic hearing may provide only limited benefit for perception of spatial cues in Bimodal CI users, Dorman et al.^21^ reported significant improvements in localization for BiEAS users. The availability of residual hearing in both ears may allow the listeners to use ITD cues, thus resulting in improved sound localization. Gifford et al.^39,40^ showed significant speech recognition and subjective perceptual benefits for BiEAS compared to the Bimodal listening condition. They also found that limiting the CI bandwidth (i.e., increasing the LCF to the CI) yielded significant improvement for speech recognition in noise and subjective estimates of listening difficulty. These data suggest that tonotopic mismatch may significantly limit the benefit of BiEAS listening, consistent with previous simulation studies showing that EAS is highly sensitive to tonotopic mismatch^36^. Tonotopic matching within the CI appears to be important for utilization of acoustic hearing in the ipsilateral ear.

Taken together, these previous studies suggest that tonotopic matching is important for Bimodal and BiEAS listening. However, most previous studies evaluated Bimodal and/or BiEAS speech performance in quiet, noise, or speech babble. The benefits of tonotopic matching on Bimodal and BiEAS listening for segregation of competing speech is not well studied. Also, the benefits of tonotopic matching may also depend on the target-masker spatial configuration. For example, Thomas et al.^34^ found limited benefit for tonotopic matching in simulations of bilateral CIs when maskers were co-located with the target speech, but a large benefit when maskers were spatially separated from the target. While tonotopic matching may be beneficial for Bimodal CI simulations when maskers are co-located with the target^37^, the benefit is less clear when the target and maskers are spatially separated. Similarly, the benefits of bilateral residual hearing for utilization of spatial cues to segregate competing speech have not been fully explored. Some studies have found BiEAS advantages over bimodal listening for spatialized noise^41–44^, but little is known regarding BiEAS advantages for competing speech, especially when target and maskers are spatially separated. Also, it is unclear how much residual hearing is needed in the implanted ear to be beneficial in conjunction with the contralateral residual hearing.

In the present study, segregation of co-located or spatially separated target and masker speech was measured in NH adults listening to acoustic simulations of Bimodal and BiEAS CI signal processing in which the frequency allocation for the simulated CI was tonotopically matched or mismatched. The first aim of the study was to evaluate bimodal perception of target speech with co-located or spatially separated maskers. We expected that speech reception thresholds (SRTs) would be higher (poorer) with spatially separated than with co-located maskers due to poorer integration of acoustic and electric hearing across ears^36^. The second aim of the study was to evaluate the effects of tonotopic matching in electric hearing on segregation of competing speech for Bimodal listening. We expected limited benefit of tonotopic matching for co-located target and masker speech, but a larger benefit when target and masker speech were spatially separated, consistent with previous bilateral CI simulation data^29^. The third aim of the study was to evaluate the effects of bilateral residual acoustic hearing (i.e., simulations of BiEAS) on segregation of competing speech. We expected limited advantage for BiEAS over Bimodal listening for co-located target and masker speech, but a larger BiEAS advantage for spatially separated target and masker speech, as the bilateral residual hearing may provide useful low-frequency binaural cues^21,44^.

## Results

**Figure 1:**
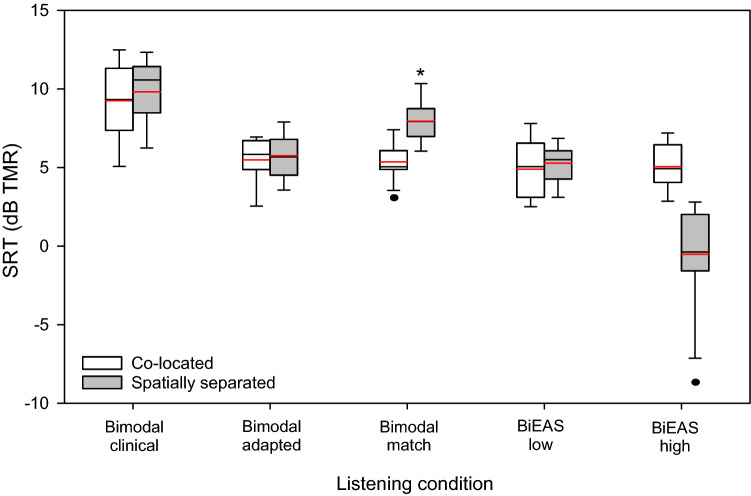
Boxplots of speech reception thresholds (SRTs) with co-located or spatially separated maskers for the five listening conditions. The boxes show the 25th and 75th percentiles, the error bars show the 10th and 90th percentiles, the circles show outliers, the black line shows the median, and the red line shows the mean.

Figure 1 shows boxplots of SRTs with co-located or spatially separated target and masker speech for the different listening conditions. Mean SRTs are shown at the top of Table 1. With the co-located maskers, SRTs were generally comparable across listening conditions except for the Bimodal clinical condition. With the spatially separated maskers, SRTs were highest (poorest) with the Bimodal clinical condition; SRTs decreased (improved) with the matched frequency allocations and the addition of ipsilateral residual acoustic hearing. A two-way repeated-measures analysis of variance (RM ANOVA) was performed on the SRT data, with listening condition (Bimodal clinical, Bimodal adapted, Bimodal match, BiEAS low, BiEAS high) and masker configuration (co-located with or spatially separated from the target) as factors; complete results are shown at the bottom of Table 1. Results showed a significant effect for listening condition [F(4, 44) = 63.2, p < 0.001], but not for masker configuration [F(1,44) = 1.5, p = 0.250]; there was a significant interaction [F(4,44) = 21.2, p < 0.001]. With the co-located maskers, post-hoc Bonferroni pairwise comparisons showed that SRTs were significantly poorer with the Bimodal clinical than with the other listening conditions (p < 0.001), with no significant difference among the remaining listening conditions. With the spatially separated maskers, SRTs were significantly higher for the Bimodal clinical and Bimodal match conditions than for the Bimodal adapted, BiEAS low, and BiEAS high conditions (p < 0.01 for all comparisons), and significantly lower for the BiEAS high condition than for the remaining conditions (p < 0.001). SRTs were significantly higher with the spatially separated than with the co-located maskers for the Bimodal match condition (p < 0.001), and significantly lower with the spatially separated than with the co-located maskers (p < 0.001) for the BiEAS high condition.

**Table 1:** Top: Mean SRTs and standard deviation for the five listening conditions with co-located or spatially separated maskers.

Masker configuration	Bimodal clinical	Bimodal adapted	Bimodal match	BiEAS low	BiEAS high
Co-located	9.25 ± 0.74	5.49 ± 0.43	5.36 ± 0.34	4.89 ± 0.55	5.06 ± 0.42
Spatially separated	9.83 ± 0.58	5.75 ± 0.42	7.95 ± 0.39	5.28 ± 0.35	− 0.51 ± 0.94

ANOVA	dF, res	F	p	η_p_^2^	Post-hoc pairwise comparisons
Condition	4, 44	63.2	< 0.001*	0.85	BC >>> BM > BA, BL >>> BH
Spatial	1,44	1.5	0.250	0.12	
Condition × spatial	4, 44	27.2	< 0.001*	0.71	BM: Spatially separated >>> Co-located BH: Co-located >>> Spatially separated Co-located: BC >> BA, BM, BL, BH Spatially separated: BC, BM >> BA, BL >>> BH

Bottom: Results of RM ANOVA on SRT data. Significant effects are indicated by italics and asterisks. Significant differences for Bonferroni-corrected pairwise comparisons are shown at right (> = p < 0.05; >>  = p < 0.01; >>>  = p <  0.001).

SRT, speech recognition threshold; BC, bimodal clinical; BA, bimodal adapted; BM, bimodal match; BL, BiEAS low; BH, BiEAS high.

**Figure 2:**
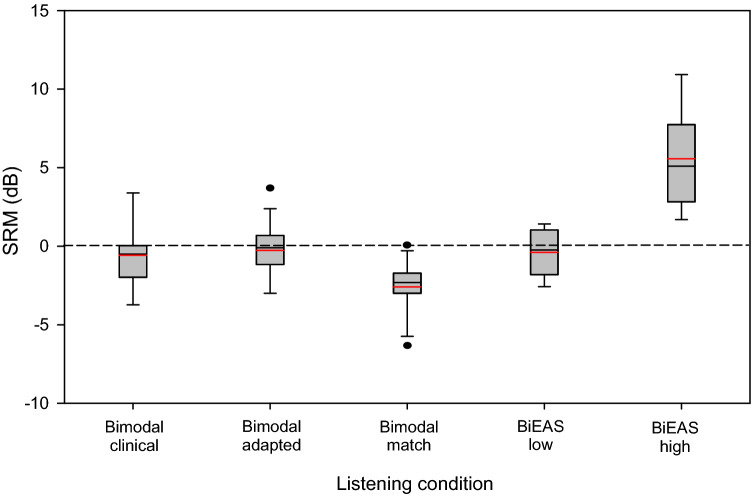
Boxplots of spatial release from masking (SRM) for the five listening conditions. The boxes show the 25th and 75th percentiles, the error bars show the 10th and 90th percentiles, the circles show outliers, the black line shows the median, and the red line shows the mean.

SRM was calculated as the difference in SRTs between the co-located and spatially separated maskers. Figure 2 shows boxplots of SRM for the different listening conditions. Mean SRM values are shown at the top of Table 2. Negative mean SRM (i.e., poorer performance with spatially separated than with co-located maskers) was observed for all listening conditions except for the BiEAS high condition. An RM ANOVA performed on the SRM data showed a significant effect for listening condition [F(4,44) = 27.2, p < 0.001]. Post-hoc Bonferroni pairwise comparisons showed that SRM was significantly higher (better) for the BiEAS high condition than for the other conditions (p < 0.01), and significantly higher for the BiEAS low condition than for the Bimodal match condition (p < 0.05).

**Table 2:** Top: Mean SRM and standard deviation for the five listening conditions.

	Bimodal clinical	Bimodal adapted	Bimodal match	BiEAS low	BiEAS high
SRM	− 0.58 ± 0.62	− 0.26 ± 0.48	− 2.59 ± 0.47	− 0.38 ± 0.41	5.57 ± 0.90

ANOVA	dF, res	F	p	η_p_^2^	Post-hoc pairwise comparisons
Condition	4, 44	27.197	< 0.001*	0.712	BH > > BC, BM, BA, BL BL > BM

Bottom: Results of RM ANOVA on the SRM data. Significant effects are indicated by italics and asterisks. Significant differences for Bonferroni-corrected pairwise comparisons are shown at right (> = p < 0.05; >>  = p < 0.01; >>>  = p < 0.001).

SRM, spatial release from masking; BC, bimodal clinical; BA, bimodal adapted; BM, bimodal match; BL, BiEAS low; BH, BiEAS high.

## Discussion

Little SRM was observed for the simulated Bimodal listening conditions, consistent with data reported in Bimodal CI simulations^45^ and real Bimodal CI users^22,23^. Tonotopic matching improved SRTs with co-located maskers, but less so with spatially separated maskers, resulting in reduced SRM. This finding is not consistent with previous data reported with simulated bilateral CIs^34^. SRM greatly improved for BiEAS listening when the amount of residual acoustic hearing was similar across ears, suggesting a great benefit for SRM with even a small amount of bilateral residual hearing (e.g., < 600 Hz). Gifford and Stecker^44^ found that the benefit of bilateral residual acoustic hearing was significantly correlated with BiEAS sensitivity to ILDs and ITDs, suggesting that SRM with BiEAS listening may be driven by perception of low-frequency spatial cues (more ITDs). The present data with the spatially separated maskers are consistent with previous studies that found significant SRM with spatialized noise for BiEAS CI simulations^42,43^. Note that mean SRM with spatialized noise was larger than with the present two-talker speech maskers (5.5 dB), possibly due to the increased susceptibility to informational masking with CI signal processing^46,47^. Combined with the spatial perception data from Dorman et al.^21^ and Gifford and Stecker^44^, the present data shows the advantage of preserving residual acoustic hearing in both ears to support segregation of spatially separated target and masker speech.

For both the Bimodal adapted and Bimodal clinical conditions, the acoustic-to-electric frequency allocation maximized the speech information delivered to the simulated CI. As with clinical fitting of Bimodal CI users, the Bimodal clinical condition introduced both an intra-aural and an inter-aural tonotopic mismatch. The Bimodal adapted condition represented long-term perceptual adaptation to this mismatch (effectively, tonotopic matching while providing the maximum speech information to the simulated CI). As such, it also represented the upper limit of Bimodal performance, assuming complete adaptation. Previous studies have shown that Bimodal CI users can partially adapt to tonotopic mismatch, but adaption is generally incomplete^48^. The present Bimodal adapted condition likely overestimated the degree of adaptation for real bimodal CI users.

If complete adaptation to the clinical frequency allocation is not possible^49^, the frequency allocation can be adjusted to match the electrode locations in the cochlea (i.e., the Bimodal match condition). In this case, the combined acoustic and electric hearing across ears would be tonotopically matched, with little spectral overlap between the stimulation modes. Interestingly, there was no significant difference in SRTs between the Bimodal match and Bimodal adapted conditions, despite the very different CI acoustic input frequency ranges. The data suggest that the loss of speech information in the CI with a tonotopically matched frequency allocation may have only limited impact on speech performance for co-located maskers, because the lost speech information in electric hearing would be represented by the residual acoustic hearing. The similarity between the Bimodal match and adapted conditions also suggests that spectral overlap may not be an issue when there is no tonotopic mismatch in electric hearing, consistent with previous studies^36,44^. Compared to the Bimodal clinical condition, in which there was a tonotopic mismatch, SRTs were significantly lower (better) with the Bimodal match and adapted conditions.

With co-located or spatially separated maskers, mean SRTs were significantly lower for the Bimodal match than for the Bimodal clinical condition. However, the mean improvement in SRTs with the spatially separated maskers in the Bimodal match condition was only 1.8 dB, half of that observed with the co-located maskers (3.9 dB). Accordingly, the mean SRM was significantly poorer for the Bimodal match (− 2.6 dB) than for the Bimodal clinical conditions (− 0.6 dB). This result is different from Thomas et al.^34^, who reported that SRM was similar for tonotopically-matched or -mismatched bilateral CI simulations. The differences in speech bandwidth between residual acoustic hearing and the CI may partly explain these different outcomes.

With the co-located maskers, mean SRTs improved from 9.3 dB with the Bimodal clinical to 5.5 dB with the Bimodal adapted condition, an improvement of 3.8 dB. Similarly, with the spatially separated maskers, mean SRTs improved from 9.8 dB with the Bimodal clinical to 5.8 dB with the Bimodal adapted condition, resulting in a similar improvement of 4.0 dB. Again, the acoustic input for the Bimodal clinical and adapted conditions maximized the speech information, with tonotopic mismatch for the clinical condition but no tonotopic mismatch for the adapted condition. While little SRM was observed for either condition, bimodal SRM was not affected by tonotopic mismatch. This suggests that Bimodal CI users may not benefit much from spatial cues even if they are able to completely adapt to tonotopic mismatch. The present Bimodal simulation data are generally consistent with previous data from Bimodal CI simulations^45^ and real Bimodal CI users^22,23^.

When there was no tonotopic mismatch, adding ipsilateral residual acoustic hearing provided no significant advantage for Bimodal SRTs when target and masker speech were co-located. This finding is not consistent with Fu et al.^36^, who found significantly better integration efficiency for vowel recognition in quiet when tonotopically matched electric hearing was combined with residual hearing in the ipsilateral rather than the contralateral ear. Differences in the speech tests and listening conditions may partly explain this discrepancy in results. A very different pattern was observed for spatially separated speech maskers. Mean SRTs improved from 8.0 dB with no ipsilateral residual hearing (Bimodal match) to 5.3 dB with ipsilateral residual hearing up to 300 Hz (BiEAS low) and to − 0.5 dB with ipsilateral residual hearing up to 600 Hz (BiEAS high). This finding is consistent with Fu et al.^36^, despite differences in speech tests and listening conditions. The improvement was likely driven by the better spatial perception with BiEAS listening^21,44^. In the present study, bilateral acoustic hearing up to 300 Hz was not tested, so it is difficult to know how the bandwidth and/or asymmetry in residual acoustic hearing may affect segregation of spatially separated target and masker speech with BiEAS listening.

There are limitations to the current study which should be noted. A critical consideration is that the tonotopic map outlined by Greenwood^50^ was based on threshold detection in NH listeners. Hearing loss and subsequent amplification by hearing aids may result in substantial distortion to Greenwood’s^50^ tonotopic map. In the present study, there was no simulation of hearing loss for the residual acoustic hearing; instead, the residual acoustic hearing was simulated by simply band-pass filtering the acoustic input between 100 and 300 Hz or 100 Hz and 600 Hz. This would not capture the degree of distortion in the residual acoustic hearing that is likely experienced by real Bimodal CI users. As such, the present simulation of residual acoustic hearing is probably a “best-case scenario” that may overestimate the contribution of residual hearing.

Another limitation is the sharp filter cutoffs in the simulated CI and residual acoustic hearing, which minimized interactions between acoustic and electric hearing within the CI ear. However, acoustic and electric stimulation may have significant overlap due to channel interaction. Gifford et al.^39^ found that greater overlap between the acoustic component and the CI allocation provided greater EAS benefit for speech performance than with the clinically recommended settings, where overlap was minimized. In the present study, the Bimodal match condition (where there was no frequency overlap between acoustic and electric hearing) produced much poorer SRM than that did the Bimodal clinical or adapted conditions. While these listening conditions were different from the EAS condition in Gifford et al.^39^, both suggest that some degree of frequency overlap between acoustic and electric hearing may benefit combined acoustic and electric hearing. Further studies are needed to better understand potential tradeoffs between tonotopic mismatch and frequency overlap between acoustic and electric hearing, within and across ears.

## Conclusions

The present simulation data suggest that hearing preservation in the implanted ear may significantly benefit Bimodal CI listeners’ utilization of spatial cues when segregating competing speech, especially when the amount of residual acoustic hearing is comparable across ears. The benefits of residual acoustic hearing in the implanted ear may be best ascertained in Bimodal CI listeners when target and masker speech are spatially separated. The present simulation data with co-located or spatially separated competing speech maskers adds to previous studies with real Bimodal and BiEAS CI users typically tested in quiet, noise, or speech babble. Bilateral low-frequency residual acoustic hearing may increase release from informational masking in CI users.

## Materials and methods

In compliance with ethical standards for human subjects, written informed consent was obtained from all participants or their legal guardians before proceeding with any of the study procedures. The study and its consent procedure were approved by the Institutional Review Board of the University of California, Los Angeles (UCLA IRB#19-000722 and IRB#18-001604) and this research was conducted in accordance with the principles of the Declaration of Helsinki and its later amendments.

### Participants

Twelve NH adults (4 males and 8 females; mean age = 35.1 years, age range: 21–64 years) participated in the study. All participants had pure tone thresholds < 25 dB HL at all audiometric frequencies between 250 and 8000 Hz. All were native speakers of American English. In compliance with the ethical standards for human participants, written informed consent was obtained from all participants before proceeding with any of the study procedures.

### Test materials

The matrix-style test materials were drawn from Sung Speech Corpus^11,12^ and consisted of a total of 50 words from five categories (Name, Verb, Number, Color, and Object), each of which contained 10 monosyllable words. Target sentences were generated by always selecting the Name “John” (the target sentence cue word), and then randomly selecting from the 10 words in each of the remaining categories. All 50 words for the target sentence were produced by a male talker and the mean fundamental frequency (F0) across all 50 words was 106 Hz. Similarly, two different masker sentences were generated by randomly selecting words from each of the categories. For each masker sentence, words were randomly selected to be different from the target sentence as well as from the other masker sentence. Thus, during each test trial, target and masker sentences were comprised of different words. All 50 words for the two masker sentences were produced by two different male talkers (mean F0s: 97 Hz and 128 Hz). A more detailed description regarding the test materials can be found in previous related studies^33,37^. The duration of the words used to generate the target and masker sentences varied slightly across categories and talkers. As such, after generating the target and masker sentences, the masker sentence duration was normalized in real-time to have the same duration as the target without affecting pitch using SoundTouch software (https://gitlab.com/soundtouch/soundtouch).

### Bimodal and BiEAS CI simulation signal processing

All target and masker stimuli were generated in real-time and delivered to circumaural headphones (Sennheiser HDA 200) via audio interface (Edirol UA-25EX) connected to a mixer (Mackie 402). Non-individualized head-related transfer functions (HRTFs) were used to create a virtual auditory space for headphone presentation of the stimuli^51^. The target sentence always originated directly in front of the listener (0° azimuth), and the two masker sentences were either co-located with the target (0°) or spatially separated from the target (± 90°).

In each test trial, the target and masker sentences were first generated according to the specified target-to-masker ratio (TMR). The TMR was calculated according to long-term root-mean-squared (RMS) power between the target sentence and each masker sentence. Note that the masker sentences were equalized in terms of long-term RMS level before the TMR calculation. The target and masker sentences were first processed by the HRTF and then mixed independently into the left and right channel. The mixed target and masker sentences in the left channel were bandpass filtered with a cutoff of 100–600 Hz and a slope of − 240 dB/octave to simulate residual acoustic hearing in the non-implanted ear. The mixed target and masker sentences in the right channel were processed by a 16-channel sine-wave vocoder to simulate electric hearing with the CI. For the sine-wave vocoder, the signal was first processed through a high-pass pre-emphasis filter with a cutoff of 1200 Hz and a slope of − 6 dB/octave. The input frequency range was then divided into 16 frequency analysis bands according to the experimental acoustic-to-electric frequency allocation using 4th-order Butterworth filters that were distributed according to Greenwood’s frequency-place formula^50^. The temporal envelope from each analysis band was extracted using half-wave rectification and low-pass filtering (cutoff frequency = 160 Hz). Next, the extracted envelopes were used to modulate the amplitude of sinewave carriers. The distribution of the carrier sinewaves assumed a 20-mm electrode array with 16 electrodes linearly spaced in terms of cochlear place. The simulated insertion depth was fixed at 24 mm, relative to the base. The frequency allocation used in the vocoder for the CI ear was manipulated to simulate three distinct speech processors:

- Bimodal clinical: The input frequency range was 200–8000 Hz while the output frequency range was upshifted to 610–11,837 Hz based on the expected spiral ganglion characteristic frequency associated with the simulated insertion depth (24 mm from the base) and electrode length (20 mm) according to the Greenwood’s function^50^. However, there was an intra-aural tonotopic mismatch within the CI ear, as well as an inter-aural low-frequency mismatch between the contralateral acoustic hearing ear and the CI ear.
- Bimodal adapted: Both the input acoustic frequency range and output frequency range were 200–8000 Hz. This bimodal condition was used to simulate long-term adaptation to tonotopic mismatch. All speech information was presented to the CI ear. Here, there was no intra-aural tonotopic mismatch within the CI ear and no inter-aural mismatch between the acoustic ear and the CI ear. Performance in this condition represented the upper limit for traditional Bimodal listening.
- Bimodal match: Both the input acoustic frequency range and output frequency range were 610–11,837 Hz. The input frequency range was tonotopically matched to the expected spiral ganglion characteristic frequency associated with the simulated insertion depth (24 mm from the base) and electrode length (20 mm), according to Greenwood’s function^50^. Low-frequency speech information below 610 Hz was truncated in the CI ear, but was preserved in the contralateral acoustic ear. Here, there was no intra-aural tonotopic mismatch within the CI ear and no inter-aural mismatch between the acoustic ear and the CI ear. There was also no frequency overlap between acoustic and electric hearing.

The mixed target and masker sentences in the right channel were also bandpass filtered to simulate the residual acoustic hearing in the implanted ear. The amount of residual hearing in the implanted ear was manipulated to simulate two additional speech processors. For both speech processors, the parametric variations in electric hearing were same as bimodal match condition.

- BiEAS low: The simulated residual hearing in the non-implanted ear was 100–600 Hz. The simulated residual hearing in the CI ear was 100–300 Hz.
- BiEAS high: The simulated residual hearing in the non-implanted and implanted ears was 100–600 Hz.

**Figure 3:**
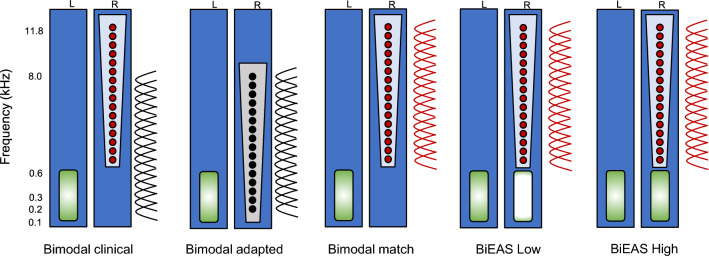
Illustration of the speech processing conditions for the left (L) and right ears (R). The green areas represent the range of simulated residual acoustic hearing. The circles represent the simulated electrode locations (output frequency range). The red circles represent a typical electrode insertion, and the black circles represent a deep insertion. The analysis bands (input frequency range) are shown at the right of each processing condition. The black analysis bands represent the clinical acoustic-to-electric frequency allocation and the red analysis bands represent a tonotopically matched allocation relative to a typical electrode insertion depth.

Figure 3 illustrates the input and output frequency bands for the left and right ears for the five listening conditions.

### Test procedures

Testing was performed in a sound-attenuating booth. SRTs were measured using an adaptive procedure (1-up/1-down) that produced a 50% correct identification of both keywords. The procedure was similar to a coordinate response matrix test^52^. Participants were instructed to listen to the target sentence (cued by the name “John”) and then click on one of the 10 response choices for each of the Number and Color categories; no other selections could be made from the remaining categories, which were greyed out. The target level was fixed at 65 dBA. The TMR was globally adjusted from trial to trial by varying the levels of each of the male maskers by the same amount according to the correctness of the response. If the participant correctly identified both the target Number and Color keywords, the TMR was reduced; if not the TMR was increased. The initial step size was 4 dB for the first two reversals in TMR, and the final step size was 2 dB. The SRT was calculated by averaging the last six reversals in TMR. The 10 listening conditions (5 listening conditions × 2 masker spatial configurations) were randomized within a test block and 2–3 blocks were tested for each participant; SRTs for each condition were averaged across blocks. Participants were given no practice or previews prior to the testing sessions, and no feedback was provided during testing. All testing was completed in a single session with short breaks between test blocks.

### Data analysis

SRT data were analyzed using an RM ANOVA, with listening condition and masker spatial configuration as within-subject factors. SRM data were analyzed using an RM ANOVA, with listening condition as the within-subject factor. Significance was defined as p < 0.05. Bonferroni correction was applied to post-hoc pairwise comparisons. RM ANOVAs were performed using SPSS (Version 20.0; Armonk, NY). All figures were generated using Sigmaplot software (Version 14).

## Supplementary Information

Supplementary Information.

## Data Availability

The raw de-identified data are included as Supplementary material (Supplementary Table S1.xlsx).
